# DRIFTS-MS Investigation of Low-Temperature CO Oxidation on Cu-Doped Manganese Oxide Prepared Using Nitrate Aerosol Decomposition

**DOI:** 10.3390/molecules28083511

**Published:** 2023-04-16

**Authors:** Xingfan Gong, Jiacheng Xu, Tiantian Zhang, Yan Sun, Shiyu Fang, Ning Li, Jiali Zhu, Zuliang Wu, Jing Li, Erhao Gao, Wei Wang, Shuiliang Yao

**Affiliations:** 1 School of Environmental Science and Engineering, Changzhou University, Changzhou 213164, China; 2 School of Material Science and Engineering, Changzhou University, Changzhou 213164, China; 3 Key Laboratory of Advanced Plasma Catalysis Engineering for China Petrochemical Industry, Changzhou 213164, China

**Keywords:** doping, Cu–Mn_2_O_4_, CO oxidation, oxygen vacancy, water resistance, DRIFTS-MS

## Abstract

Cu-doped manganese oxide (Cu–Mn_2_O_4_) prepared using aerosol decomposition was used as a CO oxidation catalyst. Cu was successfully doped into Mn_2_O_4_ due to their nitrate precursors having closed thermal decomposition properties, which ensured the atomic ratio of Cu/(Cu + Mn) in Cu–Mn_2_O_4_ close to that in their nitrate precursors. The 0.5Cu–Mn_2_O_4_ catalyst of 0.48 Cu/(Cu + Mn) atomic ratio had the best CO oxidation performance, with *T*_50_ and *T*_90_ as low as 48 and 69 °C, respectively. The 0.5Cu–Mn_2_O_4_ catalyst also had (1) a hollow sphere morphology, where the sphere wall was composed of a large number of nanospheres (about 10 nm), (2) the largest specific surface area and defects on the interfacing of the nanospheres, and (3) the highest Mn^3+^, Cu^+^, and Oads ratios, which facilitated oxygen vacancy formation, CO adsorption, and CO oxidation, respectively, yielding a synergetic effect on CO oxidation. DRIFTS-MS analysis results showed that terminal-type oxygen (M=O) and bridge-type oxygen (M-O-M) on 0.5Cu–Mn_2_O_4_ were reactive at a low temperature, resulting in-good low-temperature CO oxidation performance. Water could adsorb on 0.5Cu–Mn_2_O_4_ and inhibited M=O and M-O-M reaction with CO. Water could not inhibit O_2_ decomposition to M=O and M-O-M. The 0.5Cu–Mn_2_O_4_ catalyst had excellent water resistance at 150 °C, at which the influence of water (up to 5%) on CO oxidation could be completely eliminated.

## 1. Introduction

CO is considered to be among marked pollutants that pose a serious threat to human health and the environment, so its emission control is required [1,2]. Transition metal oxides are effective in removing CO from industrial exhaust gases. Although the study of transition metal oxides for CO catalytic oxidation has a long history [3,4,5,6,7], carrying out CO oxidation at lower temperatures is still a hot topic [8,9,10,11,12,13,14,15,16]. It has been found that cerium-based catalysts loaded with noble metals have become the most commonly used catalysts due to their high activity at low temperatures [17]. Recently, many studies focused on the active sites of cerium-based catalysts to understand the mechanism of CO oxidation [18,19,20]. There is usually H_2_O in exhaust gas, which leads to the competitive adsorption of H_2_O and CO on the active sites of the catalyst, which may change the reaction path of CO oxidation, leading to a significant reduction in CO oxidation activity [21,22]. Therefore, improving the water resistance of the catalyst is of great significance for its practical application.

Supported noble metal catalysts have good catalytic activities for CO at low temperatures, such as Au, Pt, and Ag [23,24,25]. However, due to the high price of noble metals and the current excellent performance of doped transition metal oxides in low-temperature catalytic oxidation of CO, this study focuses on developing doped transition metal oxide catalysts. Manganese oxide (MnO_2_) catalyst is highly stable, highly active, and low-cost. There are many oxygen vacancies, abundant surface hydroxyl groups, and interlayer cations on the MnO_2_ surface that are closely related to its excellent catalytic activity [26,27]. CuO has an excellent redox reactivity and high oxygen-carrying capacity [28]. Bimetallic Mn–Cu catalyst has better performance due to the synergistic effect of MnO_2_ and CuO. Xu et al. found that the reactivity of MnO_2_ could be greatly improved by Cu impregnation [29]. Wang et al. reported that CuMn_2_O_4_ showed excellent reactivity and ideal cyclic redox performance compared with single metal oxides of Mn_2_O_3_ and CuO; however, the specific mechanism of CO oxidation on CuMn_2_O_4_ is still unknown [30]. Zhang et al. studied CO oxidation on Cu/MnO_2_ and found that water can inhibit CO oxidation. They speculated that the adsorption of water on the surface of the catalyst led to the reduction of CO oxidation activity [31]. The competitive adsorption of water and CO molecules on the active sites resulted in the reduction of catalytic activity [32]. In view of the shortcomings of the existing research, it is necessary to carry out systematic experimental and theoretical research on the mechanism of CO oxidation on Cu–Mn_2_O_4_.

Kong et al. found that the formation of oxygen vacancies in oxides has a positive impact on catalytic performance [33]. Zhu et al. found that surface oxygen vacancy can be produced by vacuum annealing or H_2_ reduction, and the concentrations of Mn^4+^ to Mn^3+^ can be adjusted by changing the vacuum annealing temperature and time [34]. McFarland and Metiu reported that doping with metal ions is also one of the most effective methods for adjusting oxygen vacancy content [35].

In this study, Cu-doped Mn oxide prepared using an aerosol decomposition of copper nitrate and magnesium nitrate precursors was used as the catalyst for low-temperature CO oxidation. CO oxidation performance was evaluated using a fixed bed tube quartz reactor fed with a gas mixture containing CO with or without water. The structures and surface reactivities of the catalysts were characterized to explain the mechanisms of CO oxidation and water influence on CO oxidation.

## 2. Results and Discussion

### 2.1. Catalyst Activity

**Figure 1:**
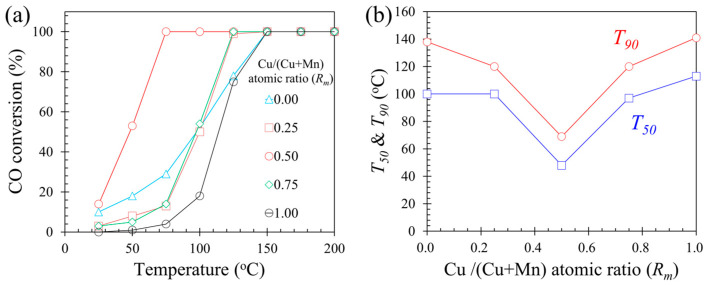
(**a**) CO conversion on each catalyst as a function of reaction temperature. (**b**) *T*_50_ and *T*_90_ as a function of Cu/(Cu + Mn) atomic ratio (*R_m_*).

Figure 1a shows the relationship between CO conversion on various catalysts and reaction temperature. With the increase in reaction temperature, CO conversion rapidly increased to 100%. The 100% CO conversion was obtained at temperatures as low as 75 °C when the atomic ratio of Cu/(Cu + Mn) (*R_m_*) was 0.5. Figure 1b illustrates *T*_50_ and *T*_90_ values at which 50% and 90% CO conversions were obtained as functions of Cu/(Cu + Mn) atomic ratio (*R_m_*). The relationship between *T*_50_ and *T*_90_ values and the Cu/(Cu + Mn) atomic ratio (*R_m_*) had a V shape. The *T*_50_ and *T*_90_ values of 0.5Cu–Mn_2_O_4_ catalyst were 48 60 °C and 60 °C, respectively. The *T*_50_ value of CO oxidation was 83 °C on 1.0% Pt/Al_2_O_3_ [16] and 80 °C on Pd_1_/CeO_2_ [18]. This fact indicates that the doped transition metal oxide catalyst (0.5Cu–Mn_2_O_4_) could also effectively achieve CO oxidation with a temperature lower than noble metal catalysts.

**Figure 2:**
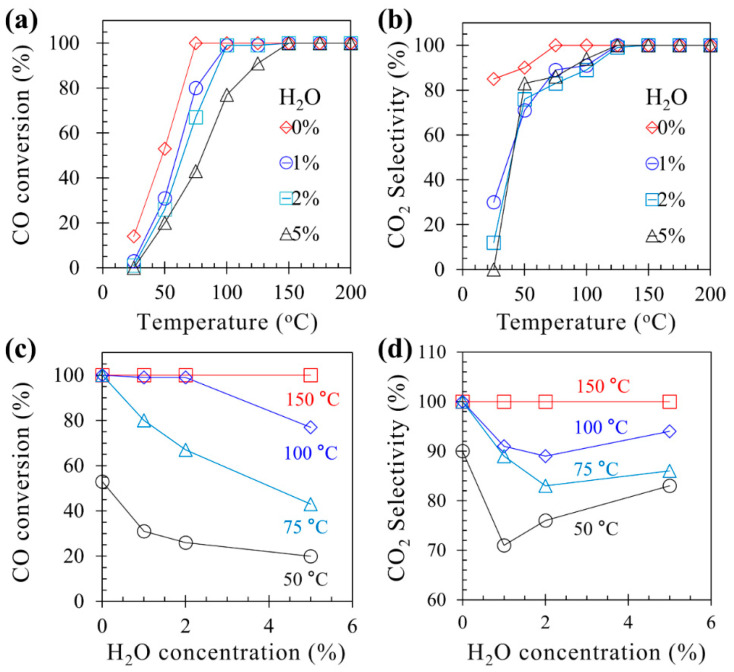
Influence of H_2_O on CO conversion (**a**,**c**) and CO_2_ selectivity (**b**,**d**).

Figure 2a–c show the effect of H_2_O (1%, 2%, or 5%) on CO oxidation on 0.5Cu–Mn_2_O_4_. CO conversion and CO_2_ selectivity decreased when increasing H_2_O concentration, indicating that H_2_O can influence CO oxidation. H_2_O (up to 5%) influence on CO conversion and CO_2_ selectivity can be eliminated at 150 °C (Figure 2d) and implies that the 0.5Cu–Mn_2_O_4_ catalyst has good water resistance performance above 150 °C.

Figure S2 shows CO oxidation during a 10 h CO oxidation duration on 0.5Cu–Mn_2_O_4_ at 150 °C. CO conversion and CO_2_ selectivity of 0.5Cu–Mn_2_O_4_ were stable in all oxidation durations, and CO_2_ selectivity was close to 100%. This finding explored that the 0.5Cu–Mn_2_O_4_ catalyst had stable water-resistance performance.

### 2.2. Structural Properties

**Figure 3:**
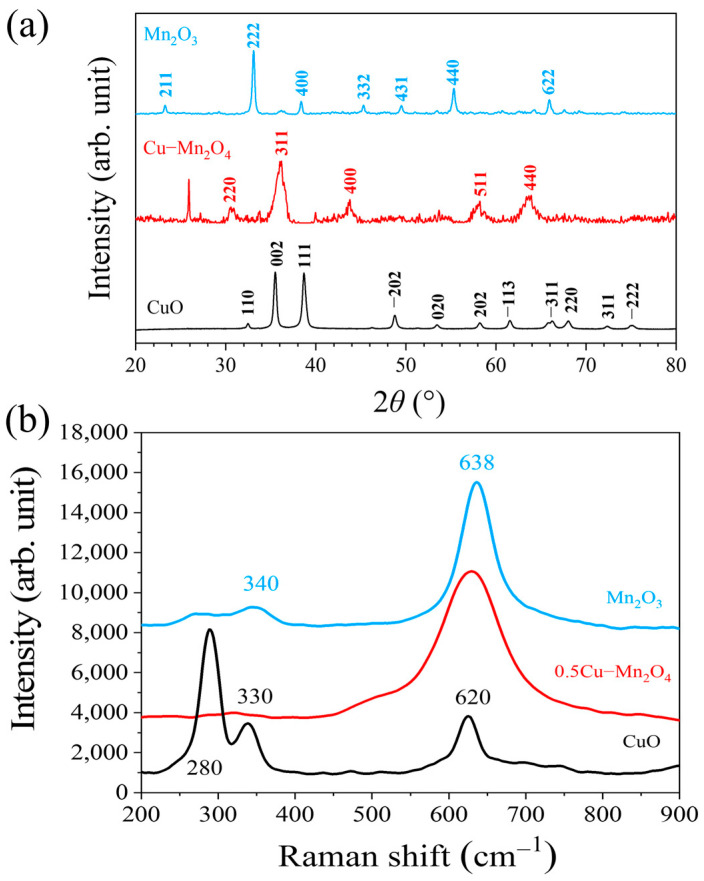
XRD patterns (**a**) and Raman spectra (**b**) of Mn_2_O_3_, 0.5Cu–Mn_2_O_4_, and CuO.

The XRD patterns of different catalysts are shown in Figure 3a. The diffraction peaks at 2*θ* = 30.4°, 37.5°, 43.5°, 57.6°, and 63.3° could be assigned to CuMn_2_O_4_ (PDF#74-2422) with spinel structure. Table 1 lists the structure parameters of Mn_2_O_3_, 0.5Cu–Mn_2_O_4_, and CuO catalysts. After Cu doping, the grain size decreased to 12.36 nm (0.5Cu–Mn_2_O_4_), significantly lower than 18.8 nm (CuO) and 27 nm (Mn_2_O_3_). The specific surface area of 0.5Cu–Mn_2_O_4_ was 56.22 m^2^ g^−1^, higher than those of Mn_2_O_3_ (24.44 m^2^ g^−1^) and CuO (15.70 m^2^ g^−1^). The peaks of 0.5Cu–Mn_2_O_4_ were flatter than those of Mn_2_O_3_ and CuO, suggesting that 0.5Cu–Mn_2_O_4_ had more defects and lower crystallinity than Mn_2_O_3_ and CuO.

Raman spectra (Figure 3b) indicates that Mn_2_O_3_ and 0.5Cu–Mn_2_O_4_ had two peaks at 638 cm^−1^ and between 300 and 340 cm^−1^. The ratio of the peak intensities at 638 cm^−1^ to that at 300–340 cm^−1^ was used to quantify the oxygen vacancy defect (OVD) ratio on Mn_2_O_3_ and 0.5Cu–Mn_2_O_4_. The OVD ratio of 0.5Cu–Mn_2_O_4_ was 16.46%, higher than the 4.35% for Mn_2_O_3_ (Table 2). Three peaks at 280, 330, and 620 cm^−1^ corresponded to the vibration modes of Ag, B1g, and B2g of CuO, respectively [36].

The N_2_ adsorption isotherms of Mn_2_O_3_, 0.5Cu–Mn_2_O_4_, and CuO are presented in Figure S3. Both Mn_2_O_3_ and 0.5Cu–Mn_2_O_4_ show Type III adsorption isotherms, while CuO shows Type IV adsorption isotherm. As listed in Table 1, the specific surface area and pore volume of 0.5Cu–Mn_2_O_4_ were higher than Mn_2_O_3_ and CuO, indicating that the Cu doping improved not only the structure but also crystallinity and defect, which could promote CO oxidation.

The metal element compositions of each catalyst were calculated using ICP-OES analysis results. The values of *R_m_*, *R_s_*-XPS, and *R_b_*-ICP are shown in Table 2. When *R_m_* was 0.5, *R_b_*-ICP and *R_s_*-XPS were 0.48 and 0.45, respectively; those three ratios were closed. The relationship of *R_b_*-ICP and *R_m_* (Figure S4) with a standard deviation (*R^2^*) of 0.9876 indicated that Cu could be successfully doped into Mn oxide with a mixing ratio of nitrate percussors. The successful doping was possibly due to the nitrate precursors of Cu and Mn being decomposed at a very closed temperature (Figure S5). For example, the temperature for 50% Mn(NO_3_)_2_ decomposition was 208 °C, close to that (245 °C) for Cu(NO_3_)_2_∙H_2_O.

**Figure 4:**
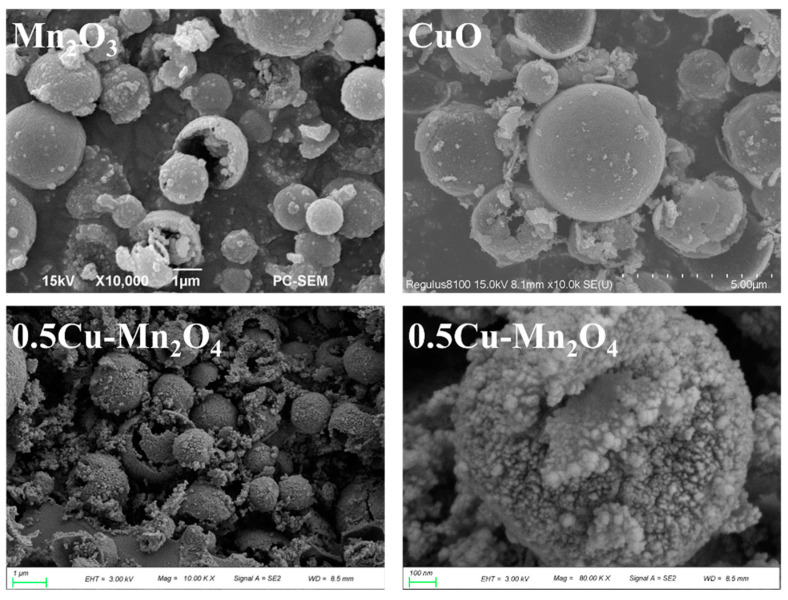
SEM morphologies of each catalyst.

Figure 4 shows the SEM images of Mn_2_O_3_, 0.5Cu–Mn_2_O_4_, and CuO. The catalysts had opened or closed hollow sphere morphologies with an outer diameter of 1–5 µm. The sphere walls of 0.5Cu–Mn_2_O_4_ were built up with many nano particles (about 10 nm), and those nano particles could be also found outside of the hollow spheres. This finding implies that the nano particles might be from the decomposition of nitrate percussor aerosols. During nitrate decomposition to oxide, NO_2_ gas was emitted, which possibly caused the nano particle accumulation to be hollow spheres.

**Figure 5:**
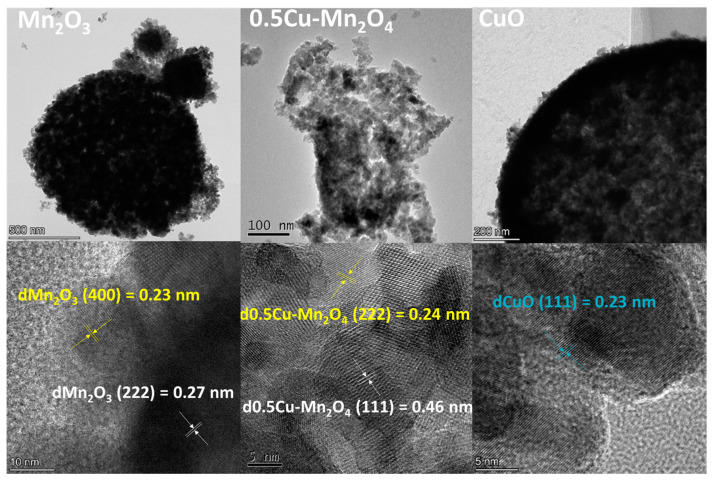
HRTEM images of each catalyst.

Figure 5 presents the HRTEM images of Mn_2_O_3_, 0.5Cu–Mn_2_O_4_, and CuO. The (400) and (222) crystal planes of Mn_2_O_3_ [37], the (311) and (111) crystal planes of 0.5Cu–Mn_2_O_4_ [38], and the (111) crystal plane of CuO [39] could be confirmed.

**Figure 6:**
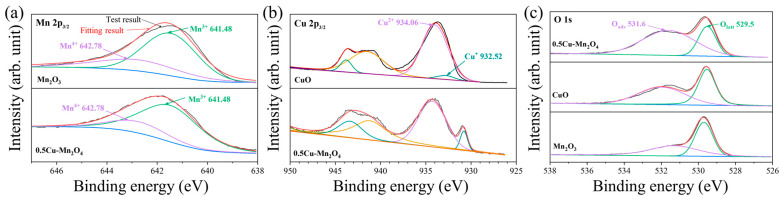
XPS spectra of Mn 2p3/2 (**a**), Cu 2p3/2 (**b**), and O1s (**c**).

The XPS spectra of Mn 2p3/2 of each catalyst are shown in Figure 6a, from which the valence state and ratio of Mn can be obtained [40]. Figure 6b shows the XPS spectra of Cu 2p3/2 of each catalyst, where the peaks at 932.52 and 934.06 eV belonged to Cu^+^ and Cu^2+^, respectively. Cu doping caused Mn^4+^ reduction to Mn^3+^, resulting in more Mn^3+^, which was conducive to the formation of oxygen vacancy defects [41]. Figure 6c shows the XPS spectra of O1s of each catalyst. The peaks of O_ads_ (531.6 eV) and O_latt_ (529.5 eV) were related to the lattice and surface adsorbed oxygen atoms, respectively [42]. Table 2 summarizes the ratios of Mn^3+^/(Mn^3+^ + Mn^4+^), Cu^+^/(Cu^+^ + Cu^2+^), and O_ads_/(O_ads_ + O_latt_). The ratio of O_ads_/(O_ads_ + O_latt_) on 0.5Cu–Mn_2_O_4_ was 72%, larger than that of CuO and Mn_2_O_3_, indicating that 0.5Cu–Mn_2_O_4_ was rich in surface adsorbed oxygen.

**Figure 7:**
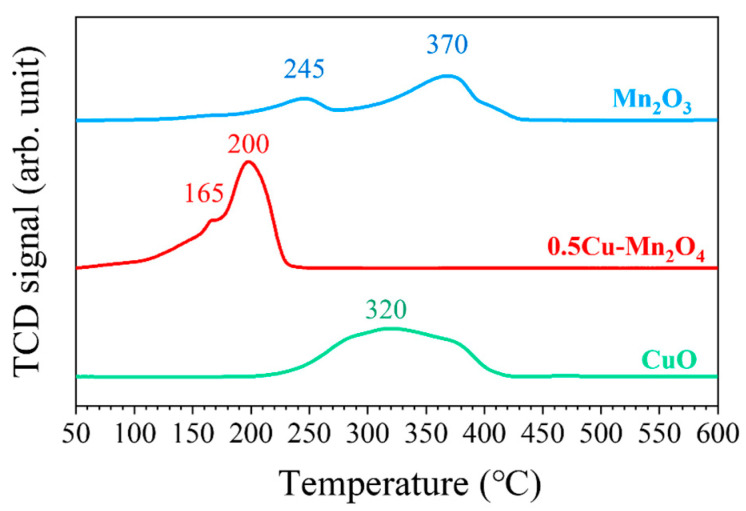
H_2_-TPR profiles.

Figure 7 shows the H_2_-TPR profiles of each catalyst. CuO had a wide peak at 320 °C, and Mn_2_O_3_ had two peaks at 245 and 370 °C. The catalyst 0.5Cu–Mn_2_O_4_ had two peaks at 165 and 200 °C. After Cu doping, the reduction peak shifted towards low temperature, indicating that a strong synergy between Mn and Cu contributed reactive oxygen species in the lattice and on the surface that could react with H_2_ at lower temperature.

### 2.3. Operando DRIFTS-MS Spectra during CO Oxidation without Water

**Figure 8:**
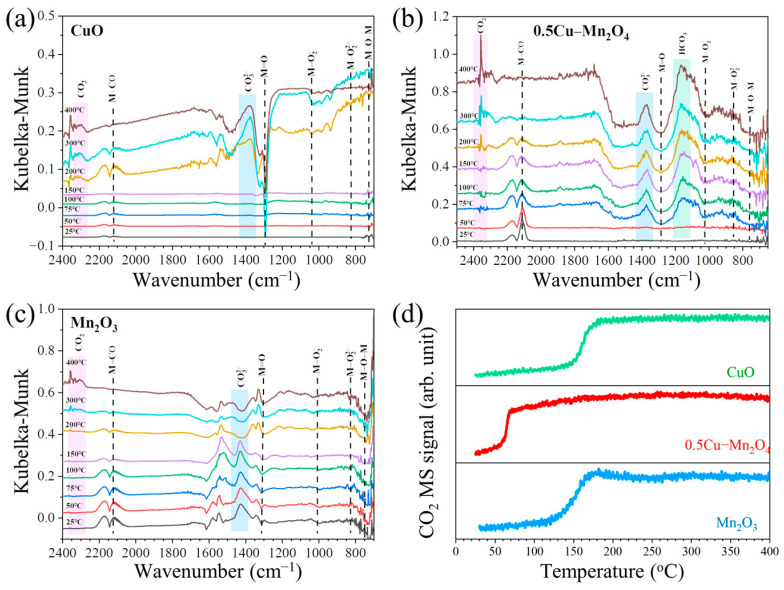
Operando DRIFTS spectra during CO oxidation between 25 and 400 °C on CuO (**a**), 0.5Cu–Mn_2_O_4_ (**b**), and Mn_2_O_3_ (**c**). (**d**) CO_2_ MS signals at various temperatures.

The DRIFTS spectra of CuO, 0.5Cu–Mn_2_O_4_, and Mn_2_O_3_ during CO oxidation from 25 °C to 400 °C with a ramp of 10 °C/min were observed (Figure 8a–c). The positive peak at 2360–2280 cm^−1^ was designated as CO_2_ [43]. The peak at 2150–2100 cm^−1^ belonged to CO adsorption (M-CO) [44]. The negative peaks at 1310–1260 cm^−1^ and 1240–1180 cm^−1^ were related to M=O (M=Cu, Mn) with terminal-type oxygen [45,46] and bicarbonate [47], respectively. The negative peak at 1022 cm^−1^ was contributed by M-O_2_^−^ [45]. The negative peaks at 770 and 840 cm^−1^ were assigned to M-O-M with bridge-type oxygen [45,46] and M-O_2_^2−^ [48], respectively. Those peaks can be clearly observed from Figure 8b. Figure 8d illustrates CO_2_ MS signals during CO oxidation on CuO, 0.5Cu–Mn_2_O_4_, and Mn_2_O_3_. It was found that CO_2_ MS signal on 0.5Cu–Mn_2_O_4_ increased when the temperature was higher than 50 °C; this temperature was obviously lower than 125 °C on Mn_2_O_3_ and 150 °C on CuO. This finding clearly shows that 0.5Cu–Mn_2_O_4_ had better low-temperature CO oxidation activity than Mn_2_O_3_ and CuO.

**Figure 9:**
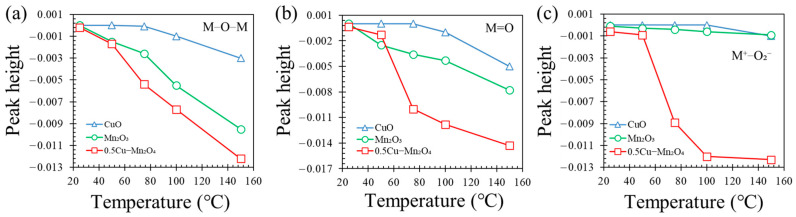
Changes of peak heights of (**a**) M-O-M, (**b**) M=O, and (**c**) M-$O_{2}^{-}$ on each catalyst as functions of temperature.

Figure 9 gives the changes in heights of the main surface oxygen-related peaks as functions of temperature. M=O and M-O-M are the main surface oxygen atoms that contributed CO oxidation; M-$O_{2}^{-}$ is a descriptor of O_2_ adsorption at M sites with oxygen vacancies [45]. M=O and M-O-M on 0.5Cu–Mn_2_O_4_ decreased more rapidly than that on Mn_2_O_3_ and CuO due to M=O and M-O-M reacting with CO. The rapid decrease in M=O and M-O-M resulted in increase in M-$O_{2}^{-}$ and formation of oxygen vacancy (M-□-M, where, the oxygen vacancy is represented by an empty square □).

### 2.4. Operando DRIFTS-MS Spectra during CO Oxidation with Water

**Figure 10:**
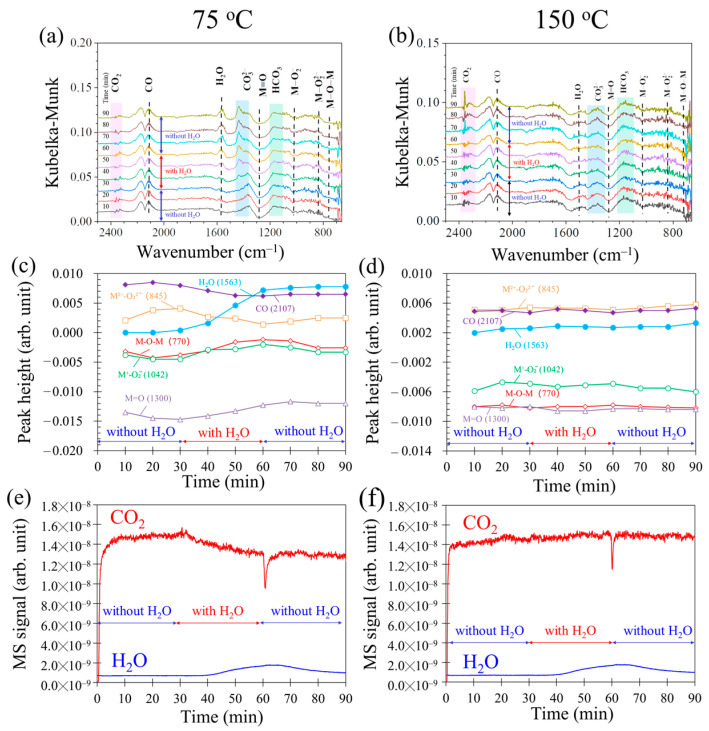
DRIFTS-MS spectra during CO oxidation with water at 75 °C (**a**,**c**,**e**) and 150 °C (**b**,**d**,**f**).

Figure 2 demonstrated that water could influence CO oxidation when the reaction temperature was lower than 150 °C. Thus, the influence process was monitored using operando DRIFTS-MS during CO oxidation with water on 0.5Cu–Mn_2_O_4_ at fixed temperatures of 75 and 150 °C (Figure 10). The monitoring experiments were carried out by feeding a gas mixture of 1% CO and 20% O_2_ (balanced with Ar) in 0–30 and 61–90 min time durations and feeding a gas mixture of 1% CO, 1% H_2_O, and 20% O_2_ (balanced with Ar) in 31–60 min time durations. Figure 10a,c,e shows DRIFTS spectra, MS signals of CO_2_ and H_2_O, and surface species, respectively, where the reaction temperature was fixed at 75 °C. It can be confirmed that the CO_2_ MS signal decreased when the gas mixture with water was supplied, indicating that H_2_O has influence on CO oxidation. This fact was consistent with the results shown in Figure 2. When water was supplied, peak heights of surface H_2_O, M=O, and M-O-M increased, while M-$O_{2}^{-}$ decreased, suggesting that H_2_O could be adsorbed on the surface and inhibited CO adsorption, resulting in M=O and M-O-M not reacting with CO. This results also indicated that O_2_ decomposition on catalyst surface was not inhibited during CO oxidation with water, as O_2_ decomposition is the process for M=O and M-O-M formations [45].

When the reaction temperature was 150 °C, no obvious changes in CO_2_ MS signal and surface oxygen-related species could be found within the 0–90 min time duration (Figure 10b,d,f), demonstrating that water had no influence on CO oxidation. This result agrees the results showed in Figure 2.

## 3. Materials and Methods

### 3.1. Catalyst Preparation

The experimental setup for catalyst preparation is shown in Figure S1. A Pyrex bottle (500 mL) was filled with a 50 mL solution of copper nitrate trihydrate (Cu(NO_3_)_2_∙3H_2_O, Sinopharm Chem. Reagent, Shanghai, China) and manganese nitrate (Mn(NO_3_)_2_, Aladdin Reagent, Shanghai, China) and equipped with an ultrasonic atomizer (ZP-01, Chenyang Electric Appliance, Zhongshan, China) that was used to generate nitrate mists. The nitrate mists together with N_2_ gas (1 L/min) were heated to 60 °C in a Teflon tube to generate nitrate aerosols that flowed into a quartz tube (12 od. × 10 id. × 350 l. mm^3^) hold at 500 °C with the use of a tubular electric furnace (KSL-6D-11, Shandong Longkou Xianke, Longkou, China). The nitrate aerosols were decomposed to Cu-doped Mn oxide and collected with two water absorption bottles. The Cu-doped Mn oxide particles were separated from the water using high-speed centrifugation (TG16-WS, Liangyou Instrument, Changzhou, China) operated at 10,000 rpm for 10 min. The precipitate of Cu-doped Mn oxide particles after centrifugation was washed with pure water 3 times and dried at 80 °C for 12 h. The atomic ratio of Cu/(Cu + Mn) (*R_m_*) was 0.00, 0.25, 0.5, 0.75, and 1.00 by controlling the amounts of manganese nitrate and copper nitrate in the solution. The obtained particle catalysts were designated Mn_2_O_3_, 0.25Cu–Mn_x_O_y_, 0.5Cu–Mn_2_O_4_, 0.75Cu–Mn_x_O_y_, and CuO, respectively.

### 3.2. Evaluation of Catalyst Activity

CO oxidation on each catalyst was evaluated using a fixed-bed tubular quartz reactor (10 od. × 4 id. × 350 l. mm^3^). The fixed bed (8 mm height) was filled with 100 mg of the catalyst. The reactor was fed with a gas mixture of 1% CO, 20% O_2_, and N_2_ balanced with a total gas flow rate of 50 mL/min with a weight hourly space velocity of 30,000 mL g^−1^ h^−1^. The concentrations of CO and CO_2_ in the gas mixture at the inlet or outlet of the reactor were analyzed online using a chromatograph (GC9790, Zhejiang Fuli, Taizhou, China).

The water resistance of the best catalyst (0.5Cu–Mn_2_O_4_) was demonstrated using a gas mixture containing 1% CO, 20% O_2_ (1%, 2%, or 5%), H_2_O, and N_2_ balance with a total gas flow rate of 50 mL min^−1^.

A 10 h long-term CO oxidation on the 0.5Cu–Mn_2_O_4_ catalyst was conducted using a fixed reaction temperature of 150 °C to evaluate water-resistance stability using a gas mixture containing 1% CO, 20% O_2_, 5% H_2_O, and N_2_ balance with a total gas flow rate of 50 mL min^−1^.

It must be noted that volumetric concentrations of all compositions in the gas mixtures were used.

### 3.3. Catalyst Characterization and Calculations

The crystalline structures were examined using an XRD (D/MAX2500, Japan) with a Cu-K_α_ radiation (*λ* = 0.15418 nm) at 40 kV and 40 mA. Raman spectra in 200–700 cm^−1^ were obtained using Horiba Scientific LabRAM HR Evolution with laser excitation of *λ* = 532 nm.

The N_2_ adsorption–desorption isotherms were collected at −196 °C using the specific surface and porosity analyzer (Autosorb-iQ2, Conta, Gainesville, GA, USA). The Brunauer–Emmett–Teller (BET) method was used to calculate the specific surface area. Pore diameter and pore volume were obtained using Barrett–Joyner–Halenda (BJH) analysis.

The metal element compositions were analyzed using the ICP-OES/MS (5110 ICP-OES, Agilent, Santa Clara, CA, USA).

The SEM and HRTEM images were taken using Regulus 8100 (Hitachi, Tokyo, Japan) and FEI Talos F200S (Thermo Scientific, St. Louis, MO, USA), respectively.

The XPS analysis was performed using Thermo Scientific K-Alpha (St. Louis, MO, USA) with Al-K_α_ X-ray radiation (*hv* = 1486.6 eV) as the excitation source. The observed elemental spectra were corrected by using a C 1s binding energy of 284.8 eV.

The H_2_-TPR measurement was carried out using an automatic temperature programmed chemistry analyzer (AutoChem II 2920, Micromeritics, Norcross, GA, USA). An amount of 30–40 mg catalyst powder was placed in a U-shaped quartz tube. The catalyst powders were pretreated from room temperature to 300 °C with a ramp of 10 °C min^−1^ in He atmosphere (50 mL min^−1^) for 1 h, cooled down to 50 °C, and supplied with 10% H_2_ (Ar balance) gas (50 mL min^−1^) for 0.5 h, heated to 700 °C with a rate of 10 °C min^−1^ in10% H_2_ (Ar balance) gas (50 mL min^−1^). The gas from the outlet of the quartz tube was detected with a thermal conductivity detector (TCD).

The DRIFTS-MS experiments were carried out as follows. The catalyst powders (about 30 mg) in the sample cell of the DRIFTS were pretreated in Ar (20 mL/min) at 300 °C for 1 h, cooled to room temperature (25 °C), and stabilized for 10 min, and then the background spectrum was collected. The sample cell was supplied with a gas mixture (1% CO, 20% O_2_, Ar balance, 20 mL min^−1^) for stabilization (20 min) and heated from 25 to 400 °C with a ramp of 10 °C min^−1^. Series software was used to collect the spectra at corresponding temperatures. Thirty-two scans were performed with a resolution of 4 cm^−1^, and the spectrum data of DRIFTS were analyzed using OMNIC software (IS 50 OMNIC 9.9 SPECTA 2.2 SERIES) during collection. The Kubelka–Munk function was used to convert the collected spectra into absorption spectra, whose intensities were linearly related to the amount of adsorbed functional group-related species. The gas from the sample cell was analyzed online using a mass spectrometer (MS) to obtain signals of CO (*m*/*z* = 28) and CO_2_ (*m*/*z* = 44).

When investigating the influence of water on CO oxidation at a fixed temperature (75 or 150 °C), water was added to the gas mixture to generate a gas mixture with water (1%H_2_O, 1% CO, 20% O_2_, Ar balance, 20 mL/min) and without water (1% CO, 20% O_2_, Ar balance, 20 mL min^−1^).

The thermal decomposition characteristics of manganese nitrate (Mn(NO_3_)_2_, Aladdin Reagent, Shanghai, China, 48.91 mg) and copper nitrate (Cu(NO_3_)_2_∙3H_2_O, 19.35 mg) in Ar atmosphere (30 mL min^−1^) were carried out by heating the nitrate from 25 to 1000 °C with a ramp of 10 °C min^−1^ using a thermogravimetric analyzer (TG, LABSYS evo TGA, Setaram, Caluire, France).

The CO conversion and CO_2_ selectivity were calculated using Equations (1) and (2), respectively.

(1) $$COconversion=\frac{{\left[CO\right]}_{0}-[CO]}{[{CO]}_{0}}\times 100\%$$

(2) $$S_{{CO}_{2}}=\frac{\left[{CO}_{2}\right]}{\left[{CO]}_{0}-[CO\right]}\times 100\%$$

where [CO]_0_ and [CO] are CO concentrations in the gas mixture from the inlet and outlet of the reactor, respectively.

Cu/(Cu + Mn) atomic ratio (*R_m_*) in the nitrate solution for atomization was defined as the moles of Cu(NO_3_)_2_ to the total moles of Cu(NO_3_)_2_ and Mn(NO_3_)_2_.

Cu/(Cu + Mn) ratio (*R_b_*-ICP) in each catalyst measured using the ICP-OES was defined using Equation (3).

(3) $$R_{b}-ICP=\frac{C_{Cu}}{C_{Cu}+C_{Mn}}$$

where *Cc_u_* and *C_Mn_* in mol/kg were obtained from ICP-OES analysis.

Cu/(Cu + Mn) ratio (*R_s_*-XPS) on the surface of each catalyst was calculated using Equation (4).

(4) $$R_{s}=\frac{W_{Cu}}{W_{Cu}+W_{Mn}}$$

where *Wc_u_* and *W_Mn_* in the atom ratio were obtained from XPS analysis.

Oxygen vacancy defect (OVD) ratio was calculated from the Raman analysis results using the intensity of the peak at 638 cm^−1^ to that between 300 and 340 cm^−1^.

## 4. Conclusions

In this study, Cu-doped manganese oxide prepared using aerosol decomposition was used as a CO oxidation catalyst. The CO-oxidation and water-resistance performance were evaluated. DRIFTS-MS was used to investigate the mechanisms of CO oxidation and water resistance. The main results are summarized as follows:

(1) The catalyst 0.5Cu–Mn_2_O_4_ of 0.48 Cu/(Cu + Mn) atomic ratio had the best CO oxidation performance. *T*_50_ and *T*_90_ were as low as 48 and 69 °C, respectively.
(2) Cu could be successfully doped into Mn_2_O_4_ due to their nitrate precursors having closed thermal decomposition properties, which ensured the atomic ratio of Cu/(Cu + Mn) close to the atomic ratio in nitrate precursors.
(3) The catalyst 0.5Cu–Mn_2_O_4_ had a hollow sphere morphology, and the sphere wall was composed of a large number of nanospheres (about 10 nm), yielding the largest specific surface area and the defects on the interfacing of the nanospheres.
(4) The catalyst 0.5Cu–Mn_2_O_4_ had high Mn^3+^, Cu^+^, and O_ads_ ratios, which facilitated oxygen vacancy formation, CO adsorption, and CO oxidation, respectively, which had a synergetic effect on CO oxidation.
(5) Terminal-type oxygen (M=O) and bridge-type oxygen (M-O-M) on 0.5Cu–Mn_2_O_4_ were reactive at a low temperature, resulting in a good low-temperature CO oxidation performance.
(6) Water could adsorb on 0.5Cu–Mn_2_O_4_ and inhibited M=O and M-O-M reaction with CO. Water could not inhibit O_2_ decomposition to M=O and M-O-M. The catalyst 0.5Cu–Mn_2_O_4_ had excellent water resistance at 150 °C at which the influence of water (up to 5%) on CO oxidation could be completely eliminated.

## Figures and Tables

**Table 1:** Specific surfaces areas and pore properties of catalysts.

Sample	Specific Surface Area (m^2^/g)	Pore Volume (cm^3^/g)	Pore Size (nm)	Grain Size (nm)
Mn_2_O_3_	24.22	0.079	14.02	27.0
0.5Cu–Mn_2_O_4_	56.22	0.228	15.79	12.4
CuO	15.70	0.050	5.82	18.8

**Table 2:** Characterization results of each catalyst using XPS, ICP and Raman.

Catalyst	O_ads_/(O_ads_ + O_latt_) (%)	Mn^3+^/(Mn^3+^ + Mn^4+^) (%)	Cu^+^/(Cu^+^ + Cu^2+^) (%)	*R_m_*	*R_b_*-ICP	*R_s_*-XPS	*OVD* (%)
Mn_2_O_3_	49	51.8		0.00	0.00	0.00	4.35
0.25Cu–Mn_x_O_y_				0.25	0.29		
0.5Cu–Mn_2_O_4_	72	81.7	9.8	0.5	0.48	0.45	16.46
0.75Cu–Mn_x_O_y_				0.75	0.94		
CuO	53		8.9	1.00	1.00	1.00	

## Data Availability

The raw data are available from the corresponding author upon reasonable request.

## Supplementary Materials

The following supporting information can be downloaded at https://www.mdpi.com/article/10.3390/molecules28083511/s1. Figure S1, Diagram of catalyst preparation system. Figure S2, CO conversion (**a**) and CO_2_ selectivity (**b**) during 10-h CO oxidation on 0.5Cu–Mn_2_O_4_. Figure S3, N_2_ adsorption isotherms of Mn_2_O_3_, 0.5Cu–Mn_2_O_4_, and CuO. Figure S4, Relationship of atomic ratios of Cu/(Cu + Mn). Figure S5, Decomposition profiles of Mn(NO_3_)_2_ and Cu(NO_3_)_2_∙H_2_O.

## Sample Availability

Samples of the catalysts are not available from the authors.
