# Supplementary material for: DRIFTS-MS Investigation of Low-Temperature CO Oxidation on Cu-Doped Manganese Oxide Prepared Using Nitrate Aerosol Decomposition
Source: Molecules. 2023 Apr 16;28(8):3511. doi: 10.3390/molecules28083511 (PMC10144047; doi:10.3390/molecules28083511)
Supplement: Supplementary file 1 [file molecules-28-03511-s001.zip › molecules-2329315-supplementary.pdf]

Supplementary Materials for

DRIFTS-MS Investigation of Low-Temperature CO Oxidation on Cu-Doped Manganese Oxide Prepared Using Nitrate Aerosol Decomposition

Xingfan Gong[1], Jiacheng Xu[1,2], Tiantian Zhang[1], Yan Sun[1], Shiyu Fang[1], Ning Li[1], Jiali Zhu[1,3], Zuliang Wu[1,3], Jing Li[1,3], Erhao Gao[1,3], Wei Wang[1,3], Shuiliang Yao[1,2,3,*]

[1] School of Environmental Science and Engineering, Changzhou University, Changzhou 213164, China
[2] School of Material Science and Engineering, Changzhou University, Changzhou 213164, China
[3] Key Laboratory of Advanced Plasma Catalysis Engineering for China Petrochemical Industry, Changzhou 213164, China
* Correspondence: yaos@cczu.edu.cn

S1. Experimental system (Figure S1)

S2. Results (Figure S2-S5)

S1. Experimental system

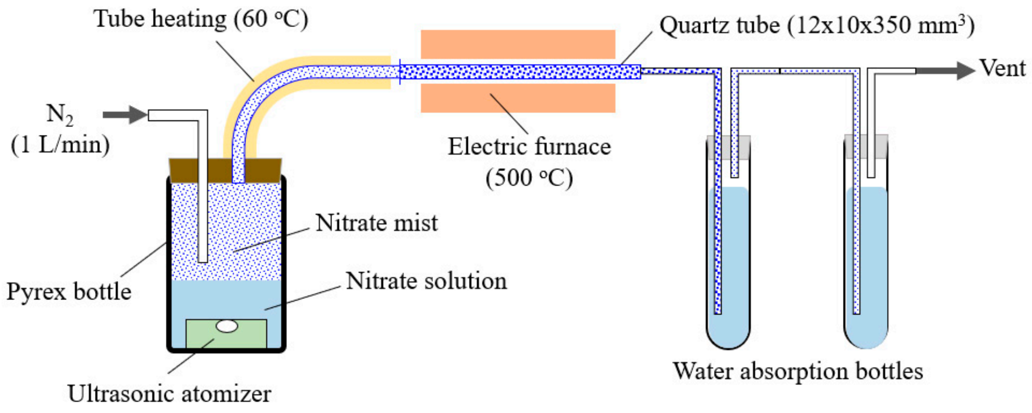

Figure S1. Diagram of catalyst preparation system.

S2. Results

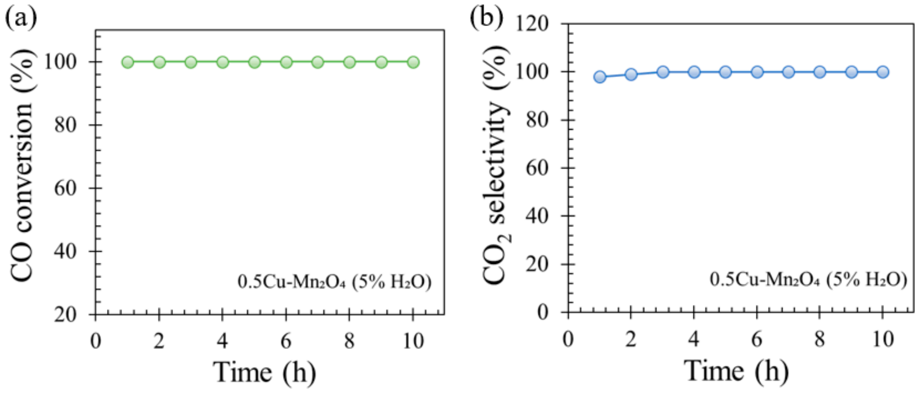

Figure S2. CO conversion (**a**) and CO_2 selectivity (**b**) during 10-h CO oxidation on 0.5Cu-Mn_2O_4.

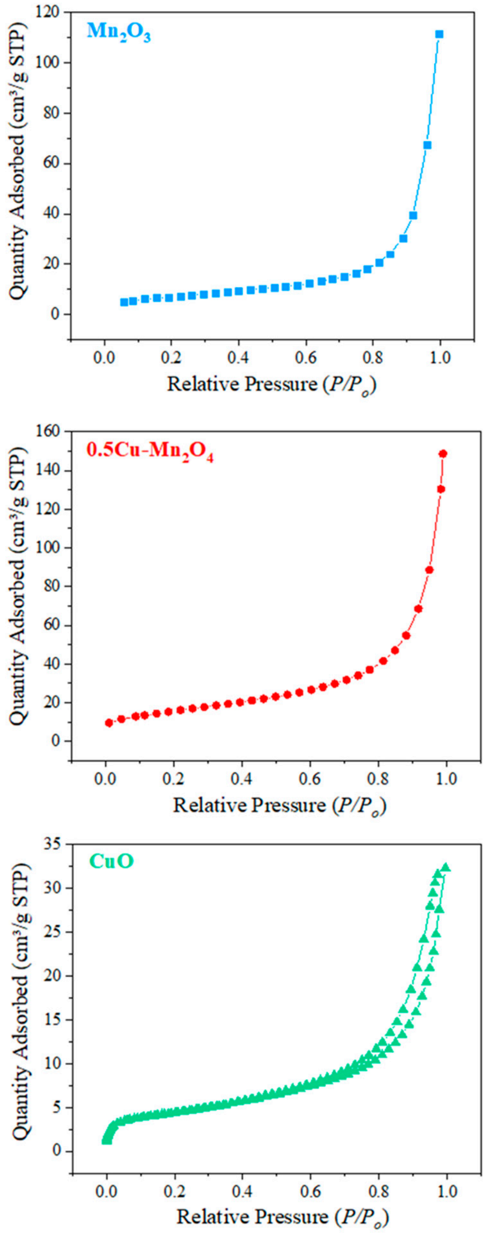

Figure S3. N_2 adsorption isotherms of Mn_2O_3, 0.5Cu-Mn_2O_4, and CuO.

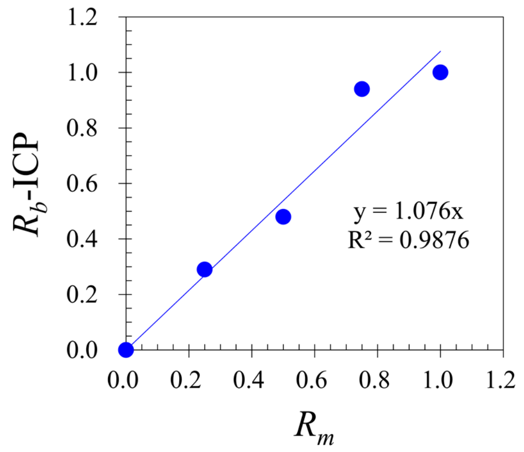

Figure S4. Relationship of atomic ratios of Cu/(Cu + Mn).

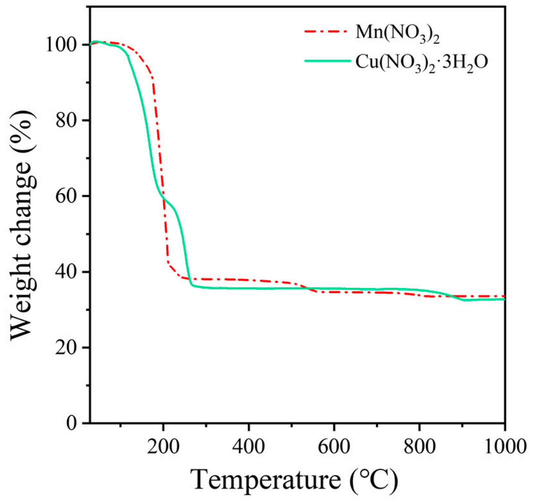

Figure S5. Decomposition profiles of $Mn(NO_3)_2$ and $Cu(NO_3)_2{\cdot}H_2O$.
